# Vitamin d deficiency impacts on expression of toll-like receptor-2 and cytokine profile: a pilot study

**DOI:** 10.1186/1479-5876-11-176

**Published:** 2013-07-22

**Authors:** Samar Ojaimi, Narelle A Skinner, Boyd JG Strauss, Vijaya Sundararajan, Ian Woolley, Kumar Visvanathan

**Affiliations:** 1 Department of Infectious Diseases, Southern Health, Clayton Rd, Clayton, Victoria, Australia; 2 Department of Medicine, Southern Clinical School, Monash University, Clayton Rd, Clayton, Victoria, Australia; 3 Departments of Infectious Diseases and Medicine, Clinical Immunology Laboratory, St Vincent’s Hospital, University of Melbourne, 4th Floor, Clinical Sciences Building, Fitzroy, Victoria 3065, Australia

**Keywords:** Vitamin D, Vitamin D deficiency, Toll-like receptor, Immunity, Innate immunity

## Abstract

**Background:**

Vitamin D is believed to play an important role outside the endocrine system in the regulation of the immune system, and in cellular proliferation and differentiation. The aim of the study was to investigate the impact of vitamin D levels on innate immunity.

**Methods:**

Participants for this prospective, longitudinal study were recruited amongst otherwise healthy staff of a large hospital in Victoria, Australia. Those fulfilling the inclusion criteria, including a vitamin D level of <50 nmol/L, were supplemented. Using flow cytometry, expression of the innate immune receptors TLR2, TLR4 and CD86 was measured on peripheral blood mononuclear cells (PBMCs) collected prior to vitamin D treatment and then at 1 and 3 months. Additonally, PBMCs at each timepoint were stimulated with specific TLR ligands and resultant supernatants were assayed for the cytokines TNFα, IL-6, IFN-α and IP-10.

**Results:**

In participants whose vitamin D level was >100 nmol/L post supplementation (n=11), TLR2 expression on PBMCs increased significantly, with no change noted in TLR4 or CD86 expression. Stimulation of vitamin D deficient samples with TLR ligands produced a number of proinflammatory cytokines, which were significantly reduced upon vitamin D normalisation. In patients whose levels returned to a deficient level at 3 months despite ongoing low-level supplementation, an increase in the pro-inflamamtory state returned. This suggests that vitamin D may play an important role in ensuring an appropriate baseline pro-inflammatory state.

**Conclusions:**

This *ex-vivo* pilot study adds clinical evidence supporting a possibly important role for vitamin D in innate immunity. If confirmed, this unique clinical study has potentially significant implications for the treatment of a variety of inflammatory conditions, where achieving optimal vitamin D levels may help reduce inflammation.

## Background

Vitamin D’s importance outside the endocrine system is being increasingly recognized, in particular in the regulation of hormone secretion and the immune system, as well as cellular proliferation and differentiation [1]. Consequently, vitamin D deficiency has been linked to an increased risk of a number of conditions, both infectious and non-infectious, including *Mycobacterium tuberculosis*, cardiovascular disease, asthma, varying malignancies and autoimmune conditions such as multiple sclerosis and type I diabetes [2-5].

The role of vitamin D with respect to adaptive immunity has been recognised for over 25 years, where it predominantly exhibits an inhibitory or suppressive effect on T-cell activation and proliferation, especially of T helper-1 cells (TH1), which produce IFN-γ and lead to activation of macrophages [1,2,6]. However, it also favours a T helper-2 (TH2) response by the regulation of the secretion of other cytokines, resulting in increased production of IL-4, IL-5 and IL-10 by T-cells [1,2,7,8].

More recently, there is evidence that vitamin D may play an important role in innate immunity. The innate immune system involves the activation of transmembrane pattern recognition receptors such as Toll-Like Receptors (TLRs) that interact with specific pathogen proteins [1,2]. This in turn leads to release of antimicrobial peptides and the subsequent killing of the organism [1,2]. TLRs are also instrumental in activating adaptive immunity [9]. TLR2 senses lipopeptides from bacteria and leads to activation of NF-κB and induction of cytokine production and release [10,11]. TLRs also lead to expression of T-cell costimulatory molecules CD80 and CD86 via induction of IFNβ [11,12]. These molecules are expressed on antigen presenting cells and are also important for B-cell activation [13]. Both TLR2 and TLR4 on monocytes have been shown to be downregulated by the immunomodulatory effect of vitamin D [14]. TLR2 has also been shown to be important in response to vaccines, with TLR2-knock out mice having reduced responses to vaccination with lipopeptides [11]. These studies prompted us to assess further the role of vitamin D on the innate immune system, specifically TLR2.

In this prospective, longitudinal pilot study, we aimed to explore whether vitamin D deficiency results in an increased innate inflammatory signature and enhanced TLR activation. No prospective data with repeated measures of vitamin D levels and markers of innate immunity are present in the published literature.

## Methods

### Study design and subjects

This study was designed as a pilot prospective longitudinal study in which otherwise healthy vitamin D deficient participants were followed over a period of three months after supplementation. Following approval from the institutional ethics committee, participants were recruited within the Southern Health Care Network, Victoria, Australia. Recruitment occurred at the end of Winter to maximize the chance of vitamin D deficiency.

After obtaining informed consent, participants went on to have further testing if they met the basic screening inclusion criteria, which included: being male (between 18 and 50 years of age) or female (>18, with menstruation within the last year) and having a Body Mass Index (BMI) between 20 and 30. Vitamin deficiency was defined as 25-OH-vitamin D levels of <50 nmol/L. Participants were also screened for conditions that may affect vitamin D metabolism and levels including a history of renal, hepatic, thyroid, parathyroid, coeliac or other malabsorptive diseases. Those with any such conditions were excluded from the study. Those with chronic diseases, or conditions which may be associated with elevated inflammatory states, were also excluded, including diabetes, chronic infections such as chronic hepatitis and Human Immunodeficiency Virus infections, autoimmune conditions, asthma or cardiovascular diseases. Participants were also screened on history for symptoms suggestive of active infection.

**Figure 1:**
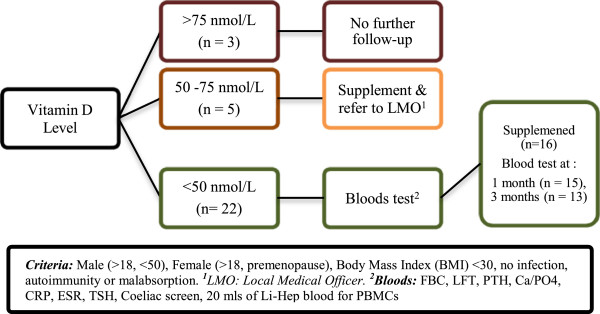
Summary of study protocol.

A summary of the study protocol is outlined in Figure 1.

### Vitamin D supplementation

Initially, all participants had baseline bloods measured. Those who were vitamin D deficient were then loaded with a single tablet of 50,000 units of vitamin D, given daily for 10 days. This formulation had not at the time been approved in Australia. However, it had received ethics approval in our hospital for use in the tuberculosis clinic and had been approved for routine use in other jurisdictions overseas. After loading, subjects went on to a maintenance regimen, which included one tablet (50,000 units) monthly. This continued for a total of 3 months of therapy, with a total of two monthly maintenance doses. After this period, with the completion of the study, participants were encouraged to continue with vitamin D supplementation at 1,000 units/day.

### Blood specimens

As outlined in Figure 1, 20 mls of blood was collected in a Lithium-heparinized tube at baseline, one-month and three-month stages. Bloods analysed at one month were taken just prior to the first monthly dose, whereas those at the three-month stage were taken 1 month after the second monthly dose. Peripheral blood mononuclear cells (PBMCs) were isolated by Ficoll-gradient centrifugation and stored in liquid nitrogen for batch stimulation and flow cytometry. Plasma was also stored at −80°C.

Participants’ vitamin D levels were measured using the Liaison 25-OH-vitamin D assay (DiaSorin SpA, Saluggia, Italy), a direct competitive chemiluminescent immunoassay.

### Laboratory analysis

#### Flow cytometry for TLR expression

Two million PBMCs were stained for flow cytometry with the following anti-human monoclonal antibodies: anti-TLR2-FITC (clone TLR2.1, eBioscience, San Diego, CA), anti-TLR4-PE (clone HTA125, eBioscience), anti-CD14-APC Cy7 (MΦP9, BD Biosciences, San Jose, CA) and anti-CD86 (clone 2331 FUN-1, BD Biosciences). Ten thousand CD14positive monocytes were acquired on a BD FACS Canto. FlowJo software (Treestar, Ashland, OR) was used for analysis and the geometric mean fluorescence of each sample was normalized to its own isotype-matched control.

#### Cytokine production following *in vitro* PBMC stimulation with TLR ligands

PBMCs were rapidly thawed and viability determined by Trypan Blue exclusion (all samples were greater than 95%). One million PBMCs were stimulated in polystyrene tubes in 1ml of RPMI-1640 supplemented with 5% Foetal Calf Serum (FCS) and penicillin / streptomycin containing either 100 ng/ml Pam3Cys (tripalmitoyl-S-glycerylcysteine, Invivogen, San Diego, CA), 10 μg/ml PolyI:C (Invivogen), 100 ng/ml LPS (lipopolysaccharide) from *Escherichia coli* strain K235 (Sigma Lifesciences), 5 μg/ml R848 (Invivogen), 0.3 μM CpG 2006 or unstimulated (media alone). After 24 hours incubation at 37°C, 5%CO_2_, 95% humidity, the supernatants were collected and stored at −20°C for cytokine ELISA.

#### Cytokine ELISA

The culture supernatants were assayed using ELISA, for TNF, IP-10, IL-6 (BD Biosciences, San Jose, CA) and IFNα (BenderMedSystems, Vienna, Austria) according to the manufacturers specifications. Assay sensitivities were 8 pg/ml for TNF, IP-10 and IFNα and 5 pg/ml for IL-6.

### Statistical methods

We first compared TLR and cytokine levels for participants who were vitamin D deficient at baseline and normalised after supplementation to levels >100 nmol/L at 1 month, (n=11). After this, TLR and cytokine levels were compared at 1 month and 3 months for those participants whose vitamin D at 3 months had returned to deficient levels (n=9). P-values were calculated using the Wilcoxon Signed Rank Test with a one-sided alpha of 0.05.

Statistical analysis was carried out using Graphpad Prism version 5 (Graphpad Software, La Holla, California).

## Results

Out of 30 healthy staff volunteers, who were a mixture of doctors, clinical and research laboratory staff, 22 (73%) had a vitamin D level of <50 nmol/L. Baseline characteristics are shown in Table 1. Of these 22 deficient participants, two were excluded due to abnormal renal function and four reversed their decision to participate. At the end of the three months, 3 participants were lost to follow up, mainly due to employment relocation and inconvenience. There were no reported adverse events as a result of supplementation.

**Table 1:** Participant characteristics and results

**Baseline characteristics**	**Baseline (n=16)**	**1 Month (n=15)**	**3 Months (n=13)**
**Mean Age (yrs)**	33.3 ± 7.4	--	--
**Sex (M:F)**	3:13	--	--
**BMI (kg/m**^**2**^**)**	23 ± 3	--	--
**Vitamin D level (nmol/L)**	28.9 ± 7.9	106.4 ± 27.0 (P<0.0001)*	77.5 ± 28.0 (P=0.0002) ^%^
**Ca (mmol/L)**	2.3 ± 0.1	2.3 ± 0.1 (P=0.76)*	2.3 ± 0.1 (P=0.85) ^%^
**PTH (pmol/L)**	5.2 ± 2.6	4.1 ± 1.7 (P=0.07)*	4.2 ± 2.0 (P=0.07) ^%^
**CRP (mg/L)**	1.6 ± 2.0	2.6 ± 6.7 (P=0.16)*	1.3 ± 2.1 (P=0.11)^%^

*PTH* – Parathyroid hormone; *Ca* – Serum Calcium; *CRP* – C-reactive Protein. PTH, Ca & CRP were measured using Beckman Coulter DXC800 Analyser (Brea, CA). Reference ranges: *PTH* – 1.7-7 pmol/L, Ca: 2.20-2.60 mmol/L ; Vitamin D 75–250 nmol/L; CRP 0–5 mg/L. *Value at 1 month compared to baseline. ^%^ at 3 month compared to baseline.

Eleven of those supplemented (61.1%) showed an excellent response with an increase in the vitamin D level to >100 nmol/L at one month. Nine of these 11 participants had returned to vitamin D levels below 100 nmol/L at 3 months, despite receiving ongoing vitamin D supplementation.

**Figure 2:**
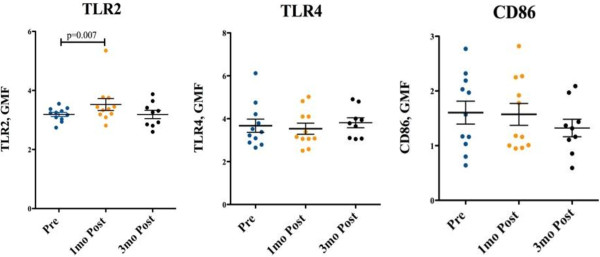
**TLR2, TLR4 and CD86 expression on Monocytes.** TLR 2, TLR4 and CD86 expression, was measured at baseline, 1 and 3 months post commencement of supplementation, in those who reached peak levels of >100 nmol/L. Geometric mean fluorescence (GMF) was measured by flow cytometry and normalised to an isotype matched control.

As seen in Figure 2, at one month, upon stimulation of PBMCs with Pam3cys there was increased expression of TLR2 in participants who reached vitamin D levels greater than 100 nmol/L upon supplementation (n=11) and was subsequently reversed at 3 months for those whose vitamin D had decreased to deficient levels (n=9). This pattern was not seen in the participants whose peak vitamin D level reached less than 100 nmol/L (data not shown). Participant numbers in this group were small, with only 3 participants following through to testing at 3 months, limiting analysis. No significant effect was seen for CD86 and TLR4 expression.

**Figure 3:**
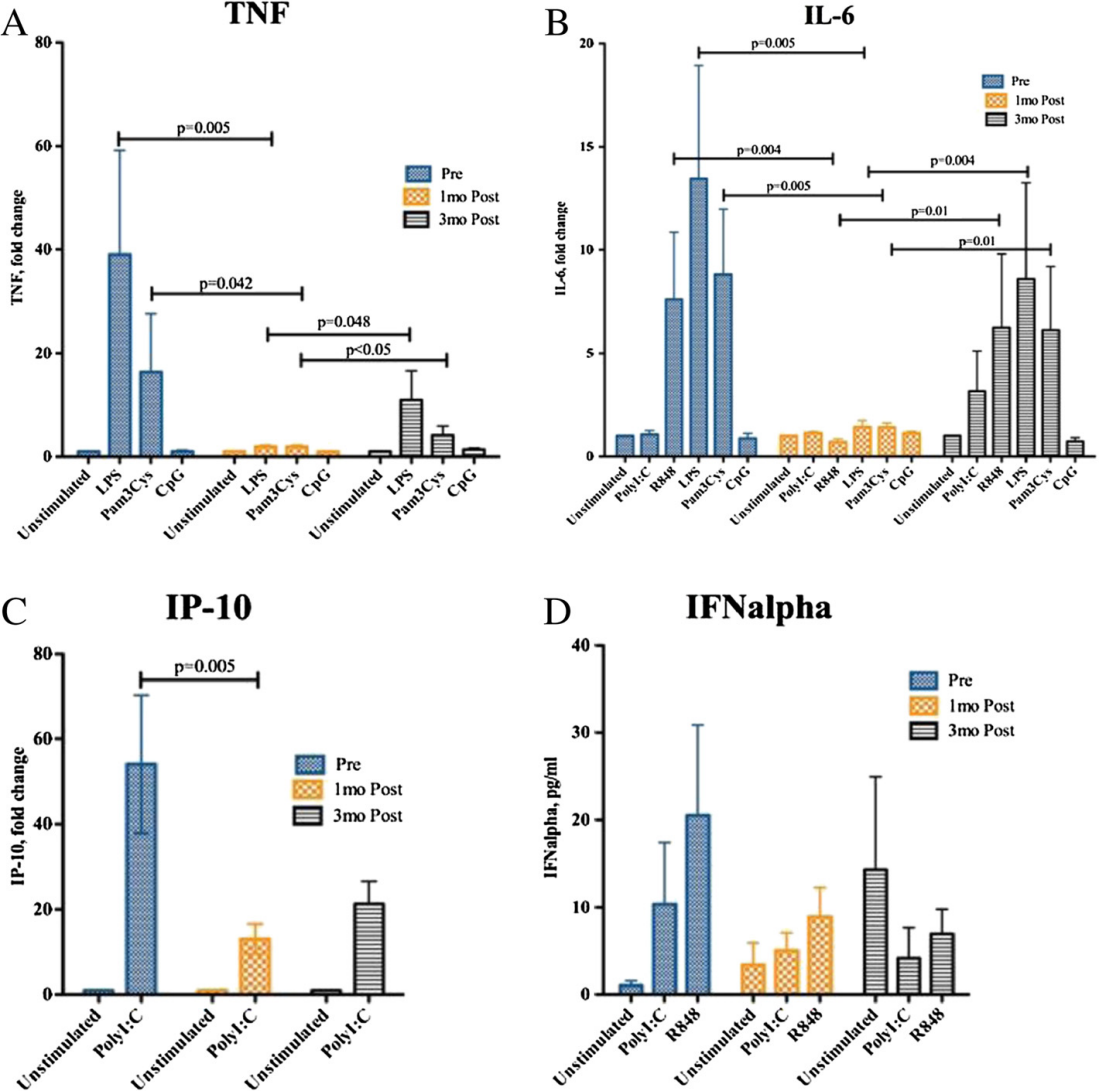
**Cytokine concentrations upon stimulation with TLR specific ligands, as measured at baseline, 1 and 3 months post start of vitamin D supplementation.** PBMCs were stimulated with TLR ligands LPS (TLR4), Pam3Cys (TLR2), CpG (TLR9) for 24 hours and supernatants were assayed by cytokine ELISA for TNF **(**Figure 3**A)** and IL-6 **(**Figure 3**B)**. Results were expressed as fold change compared to unstimulated PBMCs. In Figures 3**C** and 3**D**, PBMCs were stimulated with TLR ligands PolyI:C (TLR3) and R848 (TLR7/8) and supernatants were assayed by ELISA for IP-10 **(**Figure 3**C)** and IFNalpha **(**Figure 3**D)**. Results were expressed by fold change for Figure 3**C** and absolute values for Figure 3**D**.

Figure 3A and 3B demonstrate that, in participants with levels >100 nmol/L after one month, a statistically significant drop in TNF and IL-6 levels was seen upon TLR stimulation with LPS (TLR4 ligand) and Pam3Cys (TLR2 ligand). This indicates that, although there is no effect on TLR4 expression, vitamin D modulates the secretion of these important proinflammatory cytokines. At 3 months after the pre-load, for those with a reduction in vitamin D levels, there was a rebound rise in TNF and IL-6 levels. This drop was also seen for IP10 and IFNα, although not statistically signsificant.

## Discussion

In this small study, we have shown that supplementation of vitamin D to levels > 100 nmol/L resulted in increased expression of TLR2 on PBMCs and a reduced TLR2 stimulated cytokine profile for TNFα, IL-6 and IFN-α. Notably, this pattern reversed quickly when vitamin D levels reduced to < 100 nmol/L at 3 months after the initial load with vitamin D supplementation. TLR4 and CD86 expression were not affected by vitamin D deficiency in our study.

Vitamin D is obtained mostly via cutaneous production with ultraviolet B radiation exposure to skin [15]. It is then further metabolised in the liver and kidney into its active form 1,25(OH)_2_D [4]. In assessing vitamin D status, 25(OH)D levels are mostly measured. Levels can be affected by multiple factors, including sun exposure, dietary intake, race, age and BMI [4,16,17]. Hence, reference ranges are controversial, without an absolute consensus on normal ranges [4,16,18]. It is likely that ranges will vary for individuals with similar sun exposure, possibly relating to genetic variation in enzyme activity due to polymorphisms [15]. Vitamin D levels have also been shown to be inversely related to obesity, as measured by the BMI [17,19,20]. Some studies of highly UV-exposed adults have shown low vitamin D levels in individuals despite similar exposure, with ‘low’ levels potentially being normal for these individuals [4]. However, despite variable reports of prevalence, it has become commonly accepted that levels below 30 ng/ml (75 nmol/L) are considered to be suboptimal [4]. For calcium absorption, it is believed that a level of 80 nmol/L or higher is optimal [21], whereas for fracture risk, falls, cancer, insulin sensitivity and potentially immune function, levels as high as 120 nmol/L may be necessary [21]. One study showed improved sputum conversion and radiological appearance of tuberculosis infection in those supplemented with 0.25 mg/day (equivalent to 10,000 IU of per day) [22]. In a similar study, Martineau et al. showed the same effect only in participants with the *tt* genotype of the *TaqI* vitamin D receptor polymorphism, with the mean 25-hydroxyvitamin D level being just over 100 nmol/L [23]. In another study, respiratory infections were reduced in African American women who were receiving 2000 IU per day with serum levels up to 87 nmol/L [21]. Most experts agree, based on prior provocative testing and other studies, that vitamin D deficiency is at levels of <20 ng/mL (50 nmol/L); insufficiency, 21–29 ng/ml (50 to 75 nmol/L); the recommended level >32 ng/mL (80 nmol/L); with an upper limit of 100 ng/mL (250 nmol/L) [18,24]. Our pilot study supports levels >100 nmol/L to maintain adequate innate immune effects, as statistically significant reductions of proinflammatory cytokines and increased TLR expression upon stimulation were seen only in study participant who reached such levels. The increased TLR expression and the reciprocal reduction in the proinflammatory cytokine response were lost upon reduction of vitamin D levels back to below 100 nmol/L.

Mononuclear cells and various other cells in the body, including prostate, lung and colon cancer cells, contain 1α(OH)ase, the enzymatic machinery required to produce active vitamin D, which in turn works by binding to the vitamin D receptor (VDR), a specific nuclear receptor [8]. VDR is a ligand dependent transcription factor belonging to the superfamily of steroid, thyroid hormone and retinoid nuclear receptors [8]. Upon binding to VDR, the active form of vitamin D results in the production of cathelicidin, an antimicrobial peptide involved in the killing of mycobacteria [1]. Prior studies have shown increased TLR activation after stimulation of 1α(OH)ase and (VDR) [5,25,26]. Walker et al. showed that monocytes, derived from cord blood, exhibited decreased TLR-induced cathelicidin expression when cultured in vitamin D deficient plasma [27]. Liu et al. had previously also shown that TLR activation up-regulated VDR expression and subsequent induction of cathelicidin [26]. Lack of vitamin D, VDR or 1α(OH)ase blunts the macrophages ability to produce antimicrobial peptides [2].

While multiple studies have looked at the role of TLR2 and TLR4 activation on the expression of VDR and cathelicidin, few are available on the effect of vitamin D on TLR activation. Do et al. showed, in a cross sectional study of patients with Behçet’s disease, an inverse correlation between the expression of TLR2 and TLR4 and vitamin D levels. Additonally, in vitro, they demonstrated that vitamin D3 was able to suppress protein and mRNA expression of both TLR2 and TLR4 [9]. In contrast, our study showed an increase in TLR2 expression upon supplementation of vitamin D to levels above 100 ng/mL (250 nmol/L). This fits with the hypotheses that vitamin D is an immune modulator which varies in its effect based on the presence of inflammation. In the setting of Behçet’s, an inflammatory condition, vitamin D may act as a down regulator of inflammatory responses, resulting in reduced TLR expression. In healthy subjects, vitamin D may heighten TLR expression, in preparation for possible pathogen encounters. These hypotheses need to be investigated further, ideally in a large study which includes both healthy subjects and patients with other conditions, including chronic inflammatory conditions and chronic infections. There is recent evidence that vitamin D’s immunomodulatery effect is in part due to the presence of VDR on inflammatory cells and the ability of macrophages and dendritic cells to produce the active form of vitamin D in the presence of CYP27B1 (1αhydroxylase) [28]. In these cells however, the enzyme is stimulated by cytokines rather than parathyroid hormone, which stimulates this enzyme in the kidney [28]. Stimulation of TLR2 has been shown to result in increased expression of CYP27B1 [25].

Mahon et al. previously investigated the association between vitamin D and cytokine production while examining the effect of vitamin D supplementation in multiple sclerosis patients [26]. They found that supplementation significantly increased transforming growth factor (TFG)-β1, an anti-inflammatory cytokine, without a significant difference in TNF-α and IFNγ levels [29]. However, vitamin D levels in their subjects were below recommended levels despite supplementation [29], limiting interpretation of their results.

There are a number of limitations to our study. As a pilot study, the sample size was small. However, with repeated measures of both vitmain D levels and innate immune markers, we were able to efficiently use our data to demonstrate reversible changes in innate immunity when vitamin D levels changed from deficient to normal. In order to be able to properly assess the impact of different vitamin D serum levels and to explore further whether supplementation of the vitamin to levels >100 nmol/L is optimal, a larger study, with a similar repeated measures design which lends itself to investigating the reversability of vitamin D’s effect on TLR expression and cytokine production, is required. It would be important to objectively assess adherence to supplementation, such as pill-count or direct observed therapy protocols. Our study relied on word-of-mouth and may have thus been influenced by non-adherence. It would also be worthwhile to include patients with chronic inflammatory conditions and infections, in order to assess if vitamin D supplementation in these patients affects their cytokine profile. Another relevant question not addressed by this study is the clinical and functional significance of these findings. A larger study with longer supplementation and follow-up periods, allowing monitoring for rates infection or inflammatory conditions, may have permitted analysis of the potential clinical relevance of the demonstrated increase in expression of TLR2. This would have been made more relevant by comparison to an unsupplemented control group with variable vitamin D levels. In this study, an attempt was made to look at the functional meaning of the increased expression of TLR2 activation by looking at cytokine production. It would also be of interest to expand the study further in order to explore the molecular mechanisms behind our findings.

## Conclusions

While this is a small, pilot study, we have shown for the first time in humans that optimal vitamin D levels after supplementation may improve the expression of TLR2 and hence the body’s ability to fight infections. We have also demonstrated a marked reduction in the induced cytokine profile, specifically IL6, TNF and IFN alpha associated with higher vitamin D levels and the reversibility of the innate immune profile with decreasing levels. There are multiple implications for this, including the potential role for vitamin D to dampen down the innate immune system in autoimmune conditions. In addition, there may be a role for vitamin D as a modulator of immune response to vaccines. To help further delineate the benefits of vitamin D and its role *in vivo*, larger, randomised, double-blinded, controlled studies with broader sampling of subjects, including those with inflammatory conditions, are required.

## Abbreviations

VDR: Vitamin D receptor; TLR: Toll-Like Receptors; Vitamin D: In this manuscript, mainly used for the measured form (inactive form) 25-hydroxyvitaminD [25(OH)D]); TNF: Tissue Necrosis Factor; IL: Interleukin; PBMCs: Peripheral blood mononuclear cells; IFN: Interferon; IP: Interferon gamma-induced protein; BMI: Body Mass Index.

## Competing interests

The authors declare that they have no competing interests.

## Authors’ contributions

SO contributed to the design and coordination of the study, including the ethics submission, participant recruitement, data collection and interpretation, as well as drafting of the manuscript. NAS performed the laboratory analysis, as well as described the methods used in doing so. BJGS contributed to the design of the study. VS performed the statistical analysis. IW contributed to the design and coordination of the study. KV was general supervisor of the study, with contribution to the conception and design of the study, as well as data analysis. All authors contributed to the critical editing and revision of the manuscript. They have all read and approved the final manuscript.
